# Regional variations in the trend of iron supplementation during pregnancy and its multi-level predictors in Pakistan

**DOI:** 10.1038/s41598-025-14616-6

**Published:** 2025-08-04

**Authors:** Ruhma Shahzad, Rubeena Zakar, Hamda Shahzad, Nazoora Manal Zakar, Fiza Tariq, Razan Ahmed, Florian Fischer

**Affiliations:** 1 Department of Public Health, Institute of Social and Cultural Studies, University of the Punjab, Lahore, Pakistan; 2 Department of Pediatrics, Shalamar Teaching Hospital, Lahore, Pakistan; 3 Akhtar Saeed Medical and Dental College, Lahore, Pakistan; 4 World Food Programme, Islamabad, Pakistan; 5 Department of Oral and Maxillofacial Surgery, Ziauddin University, Karachi, Pakistan; 6 Institute of Public Health, Charité – Universitätsmedizin Berlin, Charitéplatz 1, 10117 Berlin, Germany

**Keywords:** Iron supplementation, Pregnancy, Predictor, Pakistan, Diseases, Health care

## Abstract

Iron supplementation during pregnancy is a key intervention preventing and treating iron deficiency anemia, which is associated with adverse maternal and neonatal outcomes, including severe maternal anemia, miscarriage, hemorrhage, preterm birth, and low birth weight. Despite this, a comprehensive understanding of the trends and predictors of iron supplementation across different regions and provinces in Pakistan remains limited. This study aims to assess both the temporal trends in iron supplementation among pregnant women and its multi-level determinants. This research utilizes repeated cross-sectional study design using secondary data from four waves of the Pakistan Demographic and Health Survey (PDHS; 2006–07 to 2019) to analyze the regional variations on the trend of iron supplementation. Participants included ever married women of reproductive age who have responded to the question of “uptake of iron supplementation during last pregnancy”. Various individual, community and institutional level factors from the data set of PDHS 2019 were used as independent factors to study the predictors of iron supplementation among women during pregnancy. For studying the trends, rate differences, rate ratios, changes in percentages and differences in percentages of iron supplementation during pregnancy were calculated, while for analyzing the predictors of iron supplementation, binary logistic regression models were used. There has been a 44.1% increase in iron supplementation among pregnant women nationwide, with regional increases of 61.7% in rural areas and 19.9% in urban areas, leading to a current national supplementation rate of 65.4%. Factors such as older age, rural residency, living in Sindh or Baluchistan, smoking history, higher number of pregnancies and losses, and more children born or deceased were associated with lower odds of iron supplementation(*p* < 0.005). Conversely, higher education, residency in Gilgit Baltistan, Azad Jammu and Kashmi, as well as Khyber Pakhtunkhwa, and lady health worker’s advice regarding antenatal care were the significant factors with antenatal care utilization as the strongest predictor of supplementation in both unadjusted (OR = 30.07; 95% CI: 23.55–38.40) and adjusted models (AOR = 31.29; 95% CI: 14.37–68.11). Although over half of pregnant women in the study population take iron supplements, the rate is still lower compared to many other countries. Significant regional disparities suggest the need for targeted efforts to increase supplementation rates and improve maternal health outcomes, such as increasing healthcare access in underperforming regions, expanding educational campaigns, and strengthening community-based programs to improve supplementation adherence.

## Introduction

Iron deficiency anemia is a significant global public health challenge, affecting an estimated one-third of the population with pregnant women being particularly vulnerable due to increased iron demand from physiological changes and fetal development^1–3^. Iron deficiency during pregnancy can result in adverse maternal, pregnancy, and neonate outcomes including severe maternal anemia, miscarriage, hemorrhage, preterm birth, stillbirth, low birth weight, fetal growth retardation, and adverse neurodevelopmental outcomes^4–9^. In addition to this, maternal iron deficiency is also associated with other long-term adverse outcomes including autism, schizophrenia, and abnormal brain structure among children^10^.

Notably, statistics reveal that iron deficiency anemia accounts for one-fourth of maternal deaths worldwide and approximately 120,000 deaths in low-and-middle-income countries (LMICs) annually^11–13^. In Pakistan, iron deficiency anemia among women of childbearing age is a major concern. Around 20% of non-pregnant women suffer from anemia, with prevalence rates among pregnant women reaching 76.6%. The prevalence is particularly high in the second (45.7%) and third trimesters (38.2%) compared to the first trimester (16.1%)^14,15^. Overall, 41.7% women of reproductive age in Pakistan are anemic, with more critical situation in rural areas (44.3%) compared to urban areas (40.2%)^16^. This high prevalence is largely attributed to poor dietary habits and low iron intake, exacerbated among women from lower socio-economic backgrounds^17,18^.

Iron supplementation is a widely recommended intervention to prevent and treat iron deficiency anemia. Numerous national and international guidelines, including those of the World Health Organization (WHO), advocate for routine iron supplementation during pregnancy, with dosages tailored to individual needs based on factors such as hemoglobin levels, dietary intake, and underlying medical conditions^19^.

Evidence supporting the efficacy of iron supplementation in preventing and treating iron deficiency anemia during pregnancy is well-established. Randomized controlled trials, case-control studies, and meta-analyses have consistently demonstrated improvements in maternal hemoglobin levels, reductions in the risk of anemia, and decreased incidence of adverse birth outcomes with iron supplementation^20–23^. Considering these benefits of iron supplementation during pregnancy, Pakistan has notably improved its antenatal care over the past few years by providing free iron and folic acid supplementation distribution to pregnant women throughout the pregnancy, utilizing lady health workers (LHWs)^24^. However, despite these national efforts, adherence to iron supplementation during pregnancy remains suboptimal compared to other South-Asian countries^25–27^.

Evidence suggests that iron supplementation can significantly reduce the burden of iron deficiency anemia and can avert around 12,500 disability-adjusted life years (DALYs) in Europe and 2.5 million DALYs in Africa and Southeast Asia, annually^28^. However, despite the national-level initiatives to promote iron supplementation during pregnancy and its proven benefits, adherence to prescribed regimens remains suboptimal among pregnant women. Multiple factors contribute to poor adherence, including actual and perceived side effects, forgetfulness, misconceptions about iron supplementation, cultural beliefs, and access barriers to healthcare services^24,29,30^.

In order to account fully for the benefits of iron supplementation during pregnancy, the comprehensive understanding of the trend of iron supplementation over the years with the multi-level predictors influencing iron supplementation during pregnancy is crucial for developing effective interventions. These predictors can be categorized into personal, obstetric, community and institutional-level factors^4,5,9,19^. Identifying the interplay of these multi-level predictors can provide valuable insights into improving iron supplementation adherence and addressing regional disparities in maternal health outcomes.

Previous studies have evaluated the predictors of iron supplementation among women in general and pregnant women specifically, in Pakistan and other South-Asia countries^14,31–33^. However, there is a significant gap in evaluating these predictors at multiple levels while simultaneously analyzing national trends over time. Thus, the current study aims to fill this gap by utilizing data from multiple rounds of Pakistan Demographic and Health Survey (PDHS) to assess both the temporal trends in iron supplementation among pregnant women and its multi-level determinants. By providing insights into these dynamics, the study seeks to contribute to the development of more effective iron supplementation strategies, ultimately aiming to reduce the burden of iron deficiency anemia among pregnant women.

## Methods

### Study design, setting, and data sets

The present study is based on the analysis of secondary data from the largest nationally representative Pakistan Demographic and Health Survey (PDHS) conducted to provide key demographic and health indicators in the country. While the PDHS employs a cross-sectional design, this study applies a repeated cross-sectional approach to analyze the regional variations in the trends of iron supplementation. The choice of secondary data analysis is justified as it allows for an extensive regional variation of iron supplementation trends while leveraging large-scale, high-quality data that would be otherwise challenging to collect independently. The present study utilizes data from four PDHS waves (2006–07, 2012–13, 2017–2018, and 2019) to analyze the regional variations in the trends of iron supplementation during pregnancy while the first PDHS round (1990–91) was excluded due to the absence of relevant data on iron supplementation during pregnancy. However, for analyzing the predictors of iron supplementation during pregnancy, only the dataset of PDHS 2019 was used as it provides the most comprehensive and up-to-date information on relevant multilevel predictors. The analysis includes all provinces and regions covered in the PDHS dataset: Punjab, Sindh, Khyber Pakhtunkhwa (KPK), Balochistan, Islamabad Capital Territory (ICT), Gilgit Baltistan (GB), and Azad Jammu and Kashmir (AJK).

### Study population and sample size

The sample size was determined based on the available secondary data from four rounds of the PDHS. Overall, more than ten thousand ever-married women of reproductive age (15–49 years), who are a usual resident of the selected household or have stayed in the household the night before the survey are included in each round of PDHS. However, in the sample of the present study, only those women from PDHS were selected that responded to the question of uptake of iron supplementation during their last pregnancy for all datasets. This inclusion criteria ensured that the study captures data relevant to the outcome variable of the study. The specific subset of women was chosen because iron supplementation during pregnancy is a crucial public health concern, and understanding its predictors informs maternal health interventions.

A total sample of 28,502 women from the datasets of four rounds of PDHS (2006–07, 2012–13, 2017–18 and 2019) were selected for trend analysis while a complete sample of 7,159 women from the dataset of PDHS 2019 was used for predictive modelling. This refined sample across the selected rounds, offering a substantial dataset and adequate statistical power for trend analysis and predictive modeling (Table 1). Given the large sample size (*n* = 28,502 for trend analysis; *n* = 7,159 for predictive modelling) drawn from four PDHS waves, the study is statistically powered to detect robust national trends. The PDHS datasets are nationally representative and adhere to rigorous sampling protocols, ensuring generalizability of findings.

**Table 1:** Sample.

PDHS year	Total	Excluded	Included
2006–07	10,023	4,356	5,667
2012–13	13,558	6119	7,439
2017–18	15,068	6,831	8,237
2019	15,143	7,948	7,159
Total sample	53,792	25,254	28,502

### Outcome variables

The variable of interest of the present study is iron supplementation during pregnancy. We focus on the variable indicating whether respondents received or purchased iron tablets and syrup during their last pregnancy, to trace the evolution of iron supplementation practices. Specifically, from the dataset of PDHS 2006–07, 2012–13, and 2017–18, the variable M45 inquiring about “receiving or buying iron tablets and iron syrup during last pregnancy” is used whereas from the dataset of PDHS 2019, the variable Q419 inquiring about the “supplementation of iron tablets during last pregnancy” is used. The response of “Do not know” is treated as a missing response for all variables.

### Predictor variables

**Fig. 1:**
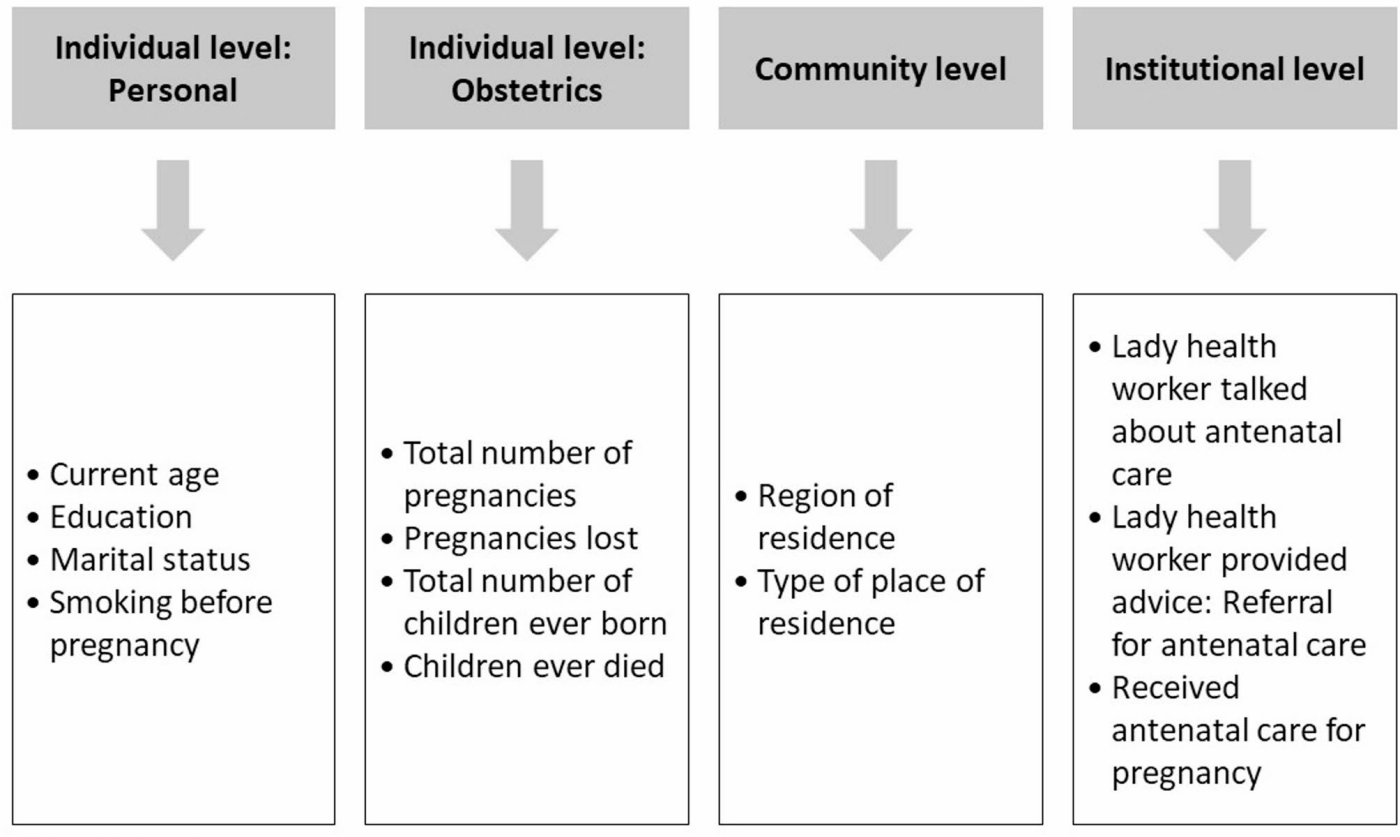
Study variables.

The study variables were chosen based on an exhaustive literature review^4,5,9,14,19,31–33^, adhering to the availability of data in the most recent PDHS round (2019). These variables were stratified into individual (personal and obstetrics), community, and institutional levels to facilitate a nuanced analysis of influencing factors. The individual level personal characteristics included the personal attributes of the females; individual level obstetrics included the reproductive history of the females; the community level variables comprised of the variables that provide insights into the broader social environment; while the institutional levels factors focused on the interactions of the females with the healthcare institute and the uptake of healthcare services. The specific variables included in the present study along with their categorization is illustrated in Fig. 1.

Data on other variables including respondent’s occupation, husband’s education, husband’s occupation, age at marriage, age at first pregnancy, and questions pertaining to female autonomy were unavailable in PDHS (2019) as it was a mini PDHS and therefore, were not included in the present study.

### Data analysis

For data analysis, Statistical Package for Social Sciences (SPSS) version 22 and Microsoft Excel 365 were used. For studying the trend of iron supplementation during pregnancy over the years, region-wise and overall percentages of iron supplementation were computed and presented graphically. Further, region and type of residence-wise rate difference, rate ratio, change in percentage and difference in percentage of iron supplementation during pregnancy were also calculated. Rate difference (highest value – lowest value) and rate ratio (highest value/lowest value) were calculated for the overall type of residence and region of residence for each year. Differences in percentage and change in percentage were calculated for each type of residence (rural/urban) and region of residence over the years. For calculating change in percentage and difference in percentage following formulas are used:

$$\:change\:in\:percentage=\:\frac{|starting\:value-final\:value|}{\left|starting\:value\right|}\:x\:100$$

$$\:difference\:in\:percentage=final\:value-starting\:value$$

To calculate the percentage change in iron supplementation during pregnancy, the starting value was taken from the PDHS 2006–07 dataset and the final value from the PDHS 2019 dataset. However, for some regions such as AJK, GB, and ICT, data were not available in the 2006–07 PDHS due to their non-inclusion in that survey. These regions were included in later surveys and were analyzed accordingly. Additionally, since data for the ICT region was not available in the PDHS 2019 dataset, the final value for ICT was taken from the PDHS 2017–18 dataset. All rates, ratios, and percentages used for the trend analysis were calculated using Microsoft Excel 365.

Further, for investigating the predictors of iron supplementation during pregnancy, Pearson’s Chi-square test was used. The associations that were found significant through Pearson’s Chi-square at p value < 0.05 were further tested using binary logistic regression to calculate odd ratios (ORs) and adjusted odd ratios (AOR). The binary logistic regression was applied hierarchically in five steps (five models) using, which are defied as follows:

1. **Model 0**: Unadjusted OR estimation.
2. **Model 1**: Adjusted for individual-level personal variables.
3. **Model 2**: Adjusted for individual-level personal and obstetric variables.
4. **Model 3**: Adjusted for individual-level personal and obstetric, and community-level variables.
5. **Model 4**: Fully adjusted model incorporating all levels, including institutional factors.

Hierarchical logistic regression is chosen to systematically assess the contribution of different predictor levels, ensuring that higher-level factors (community and institutional) are evaluated while controlling for individual characteristics. This approach also accounts for nested relationships between predictors and avoids overfitting. Before conducting regression analyses, multicollinearity and intra class analysis was assessed to ensure predictor variables were not highly correlated and to assess the model fit for regression model. Multicollinearity diagnostics indicated no significant issues; all predictors had tolerance values above 0.5 and Variance Inflation Factors (VIFs) below 2, confirming model stability. Akaike Information Criteria (AIC) and Bayesian Information Criteria (BIC) consistently decrease from Model 0 to Model 4, indicating improved model fit as more predictors are added. Model 4 has the lowest AIC/BIC and highest accuracy (78.3%), indicating the best-fitting model. The substantial drop from Model 0 (AIC = 31.260) to Model 4 (AIC = 1.827) provides very strong evidence for including individual-level personal and obstetric, community-level and institutional-level predictors (Table 2).

**Table 2:** Intra class and multicollinearityanalysis.

Model		AIC	BIC	Accuracy (f)	Tolerance	VIF
0	Unadjusted ORs	31260.915	31267.790	66.1	-	-
1	Age	16961.814	16968.025	75.1	0.991	1.009
	Education				0.991	1.009
2	Age	5825.867	5831.045	66.0	0.759	1.318
	Education				0.894	1.118
	Smoking before pregnancy				0.997	1.003
	Total number of pregnancies				0.559	1.790
	Pregnancy lost				0.862	1.160
	Children Ever Born				0.600	1.668
	Children ever died				0.894	1.118
3	Age	5960.298	5965.472	65.6	0.755	1.324
	Education				0.874	1.144
	Smoking before pregnancy				0.996	1.004
	Total number of pregnancies				0.558	1.793
	Pregnancy lost				0.860	1.162
	Children Ever Born				0.598	1.672
	Children ever died				0.894	1.119
	Region of residence				0.978	1.023
	Type of place of residence				0.967	1.034
4	Age	1827.668	1831.667	78.3	0.774	1.291
	Education				0.881	1.135
	Smoking before pregnancy				0.969	1.032
	Total number of pregnancies				0.566	1.766
	Pregnancy lost				0.884	1.131
	Children Ever Born				0.608	1.645
	Children ever died				0.882	1.133
	Region of residence				0.899	1.112
	Type of place of residence				0.940	1.063
	LHW talked about antenatal care				0.753	1.329
	LHW provided advice: Referral for antenatal care				0.750	1.333
	Received antenatal care for pregnancy				0.964	1.037

The Pearson’s Chi-square test and the hierarchal regression model are applied using SPSS version 22. All tests use *p* < 0.05 as significant value and describe the respective 95% confidence intervals (CI).

## Results

### Trend of iron supplementation during pregnancy

**Fig. 2:**
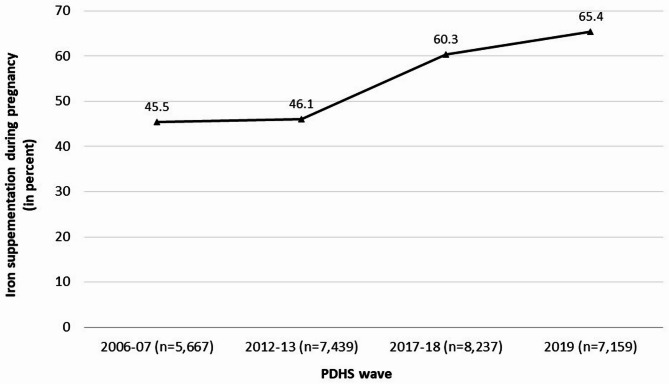
Iron supplementation during pregnancy from 2006–07 to 2019.

In PDHS 2006–07, less than half of the women reported using iron supplementation during pregnancy (45.4%) with a slight increase for 2012–13 (46.1%). However, a sudden increase was observed for PDHS 2017–18 with more than half of the women who reported using iron supplementation during pregnancy (60.3%) that increased to 65.4% for the year 2019. This illustrates an increase of 44.1% in iron supplementation during pregnancy among females of reproductive age over the years in Pakistan, equivalent to 20% points difference from 2006 to 07 till 2019 (2006–07 = 45.4% vs. 2019 = 65.4%) (Fig. 2).

### Region-wise distribution of iron supplementation during pregnancy

Concerning the rural and urban distribution of iron supplementation among females during pregnancy in Pakistan, the data of the four waves of PDHS shows a gradual increase. The prevalence of iron supplementation among women in rural areas of Pakistan is recorded to be 37.9% for 2006–07, 38.7% for 2012–13, 54.2% for 2017–18, and 61.3% for 2019.

**Fig. 3:**
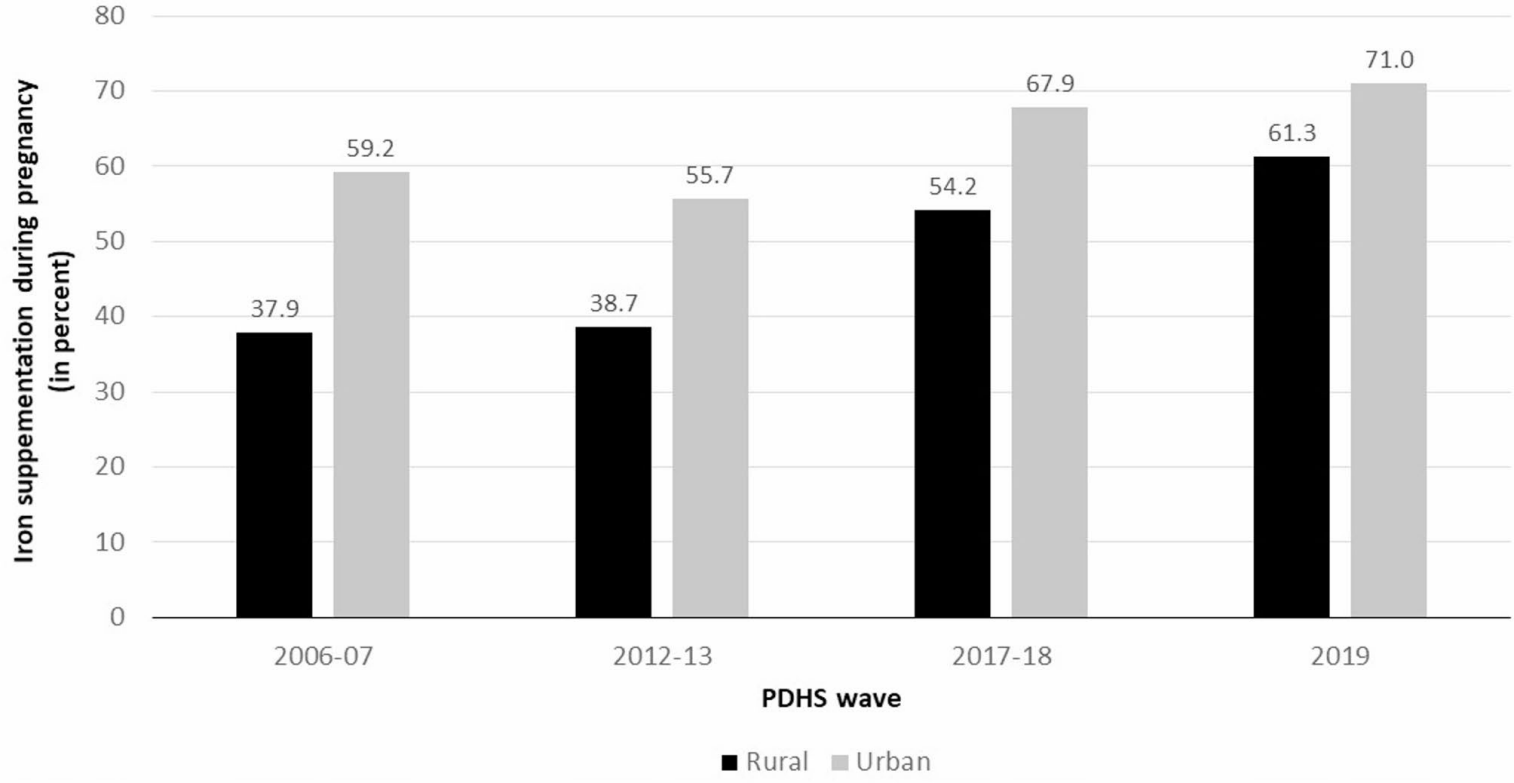
Rural and urban distribution of iron supplementation during pregnancy.

However, a higher frequency of iron supplementation in urban areas has been observed as compared to rural areas (2006–07 = 59.2% vs. 37.9%; 2012–13 = 55.7% vs. 38.7%; 2017–18 = 67.9% vs. 54.2%; 2019 = 71.0% vs. 61.3%) (Fig. 3; Table 3). Nonetheless, a decreasing trend in the rate difference and rate ratio has been observed with regard to rural and urban distribution of iron supplementation among women during pregnancy. Analysis reveals a rate difference and rate ratio among rural and urban regions of 21.3% and 1.6 for 2006–07 respectively that decreased to 17.0% and 1.4 for 2012–13; 13.7% and 1.3 for 2012–13; and 9.7% and 1.2 for 2019. These statistics show the gap of urban-rural disparities concerning iron supplementation has been covered over the years. Overall, all these statistics subject to 61.7 and 19.9% increase from 2006 to 07 to 2019 in iron supplementation during pregnancy among women of reproductive age in rural and urban areas of Pakistan respectively (Table 3).

**Table 3:** Region-wise distribution of iron supplementation during pregnancy among women of reproductive age with their rate difference, rate ratio, percentage increase, and percentage difference from the data obtained from four sets of PDHS.

	2006–07		2012–13		2017–2018		2019		% increase	% difference
	*n*	%	*n*	%	*n*	%	*n*	%		
**Type of place of residence**										
Rural	1,398	37.9	1,614	38.7	2,452	54.2	2,537	61.3	61.7	23.4
Urban	1,172	59.2	1,818	55.7	2,519	67.9	2,519	71.0	19.9	11.8
Rate difference	21.3		17.0		13.7		9.7		-	-
Rate ratio	1.6		1.4		1.3		1.2		-	-
**Region of residence**										
Punjab	950	41.6	924	46.3	1076	62.1	1297	66.4	59.6	24.8
Sindh	873	54.1	816	51.4	873	59.3	812	59.5	9.9	5.4
KPK	518	47.0	838	54.8	807	58.4	1084	72.6	54.5	25.6
Balochistan	229	34.2	237	20.6	441	45.3	456	47.3	38.3	13.1
GB	-	-	260	36.7	399	65.4	455	70.8	92.9	34.1
ICT	-	-	357	76.0	421	77.1	-	-	1.4	1.1
AJK	-	-	-	-	606	69.7	575	77.9	11.8	8.2
Rate difference	19.9		55.4		31.8		30.6		-	-
Rate ratio	1.6		3.7		1.7		1.6		-	-

With regard to the region of residence, the highest iron supplementation consumption among women during pregnancy was observed among women of Sindh in 2006–07 with more than half of the women having iron supplementation during pregnancy in the region (54.1%). This reduced to 51.4% for the year 2012–2013, increased to 59.3% in 2017–18, and remained at 59.5% in 2019 with a 9.9% increase and 5.4% difference for Sindh region from 2006 to 07 to 2019. Contrary to this, the lowest consumption of iron supplementation during pregnancy for the year 2006-07 was observed for the women residing in Baluchistan (34.2%) that further declined to 20.6% in 2012–13, doubled in 2017–18 (45.3%), and remained at 47.3% in 2019 with a 38.3% increase and 13.1% difference since 2006–07 (Table 3).

Further, a gradual increasing trend was observed in iron supplementation during pregnancy among women for other regions of the Pakistan including Punjab (2006–07 = 41.6%; 2012–13 = 46.3%; 2017–18 = 62.1%; 2019 = 66.4%), Khyber Pakhtunkhwa (KPK) (2006–07 = 47.0%; 2012–13 = 54.8%; 2017–18 = 58.4%; 2019 = 72.6%), Gilgit Baltistan (GB) (2012–13 = 36.7%; 2017–18 = 65.4%; 2019 = 70.8%), Islamabad Capital Territory (ICT) (2012–13 = 76.0%; 2017–18 = 77.1%) and Azad Jammu Kashmir (AJK) (2017–18 = 69.7%; 2019 = 77.9%). The highest percentage increase and percentage difference since 2006–07 in iron supplementation during pregnancy among women of reproductive age over the years has been observed among the women of GB (% increase = 92.9%; % difference = 34.1%), followed by Punjab (% increase = 59.6%; % difference = 24.8%), and KPK (% increase = 54.5%; % difference = 25.6%) (Table 3).

The rate difference of iron supplementation during pregnancy among the regions was 19.9% for the year 2006–07. It increased to 55.4% in 2012–13 and then suddenly decreased to 31.8% for 2017–18, and 30.6% in 2019. However, these values still illustrate a wide gap of disparities among the regions with regard to iron supplementation during pregnancy coverage. Likewise, the rate ratio was 1.6 among the regions for 2006–07 that doubled for the year 2012–13, then halved for 2017–18, and remained at 1.6 in 2019 with the highest consumption of iron supplementation for the year 2019 recorded among the women residing in AJK (77.9%) followed by KPK (72.6%), GB (70.8%), and Punjab (66.4%), while the lowest trend can be observed among the women of Baluchistan (47.3%) (Table 3).

### Socio-demographic characteristics of women who took iron supplementation during pregnancy and their associations (PDHS 2019)

In total, 7,159 women of reproductive age were analyzed that responded to the question of taking iron supplementation during their last pregnancy in PDHS 2019. Results showed that most women were in the age group of 26–35 years (49.6%) with significantly less women of age group 36 and above years reported to take iron supplementation during their last pregnancy (15.5%; *p* < 0.001). A little higher than one-fourth of the women reported having primary level of education (27.9%), while less than one-fourth of the women reported having secondary education (23.0%), with women having secondary (23.6%), higher secondary (12.6%), and higher education (19.8%) significantly reported to take iron supplementations during their last pregnancy (*p* < 0.001). A significant difference in iron supplementation among women of reproductive age was observed across provinces (*p* < 0.001) (Table 4).

**Table 4:** Socio-demographic characteristics of women taking iron supplementation during pregnancy (*n* = 7,159).

Variables	Yes		No		Total		*p*-value
	*n*	%	*n*	%	*n*	%	
	4,679	65.4	2,480	34.6	7,159	100	
**Age (n = 7,159)**							
Under 25 years	1,590	34.0	771	31.1	2,361	33.0	< 0.001***
26–35 years	2,362	50.5	1,188	47.9	3,550	49.6	
36 years and above	727	15.5	521	21.0	1,248	17.4	
**Education (n = 3,694)**							
Less than primary education	68	2.5	33	3.5	101	2.7	< 0.001***
Primary education	682	24.6	349	37.8	1,031	27.9	
Middle education	469	16.9	163	17.7	632	17.1	
Secondary education	655	23.6	196	21.2	851	23.0	
Higher secondary	348	12.6	90	9.8	438	11.9	
Higher education	549	19.8	92	10.0	641	17.4	
**Marital status (n = 7,159)**							
Currently married	4,644	99.3	2,456	99.0	7,100	99.2	0.328
Currently not married	35	0.7	24	1.0	59	0.8	
**Region of residence (n = 7,159)**							
Punjab	1,297	27.7	657	26.5	1,954	27.2	< 0.001***
Sindh	812	17.4	553	22.3	1,365	19.1	
KPK	1,084	23.2	410	16.5	1,494	20.9	
Baluchistan	456	9.7	509	20.5	965	13.5	
GB	455	9.7	188	7.6	643	9.0	
AJK	575	12.3	163	6.6	738	10.3	
**Type of place of residence (n = 7,159)**							
Urban	2,142	45.8	875	35.3	3,017	42.1	< 0.001***
Rural	2,537	54.2	1,605	64.7	4,142	57.9	
**Smoking before pregnancy (n = 7,159)**							
No	4,609	98.5	2,406	97.0	7,043	98.0	< 0.001***
Yes	70	1.5	74	3.0	144	2.0	
**Total number of pregnancies (n = 7,159)**							
Less than 4	2,569	55.5	1,088	43.9	3,657	51.5	< 0.001***
4 and above	2,083	44.5	1,392	56.1	3,475	48.5	
**Pregnancies lost**							
Yes	1,539	32.9	1,040	41.9	2,579	36.0	< 0.001***
No	3,140	67.1	1,440	58.1	4,580	64.0	
**Total number of children ever born (n = 7,159)**							
Less than 4	2,977	63.6	1,299	52.4	4,276	59.7	< 0.001***
4 and above	1,702	36.4	1,181	47.6	2,883	40.3	
**Children ever died (n = 7,159)**							
Yes	849	18.1	581	23.4	1,430	20.0	< 0.001***
No	3,830	81.9	1,899	76.6	5,729	80.0	
**LHW talked about antenatal care (n = 1,753)**							
Yes	821	62.5	225	51.3	1,046	59.7	< 0.001***
No	493	37.5	214	48.7	707	40.3	
**LHW provided advice: Referral for antenatal care (n = 1,753)**							
Yes	442	33.6	113	25.7	555	31.7	< 0.005***
No	872	66.4	326	74.3	1,198	68.3	
**Received antenatal care for pregnancy (n = 7,159)**							
Yes	4,605	98.4	1,672	67.4	6,277	87.7	< 0.001***
No	74	1.6	808	32.6	882	12.3	
* p-value calculated through Pearson’s Chi-square test ** significant at 95% level *** highly significant at 99% level							

Further, most of the women were currently married (99.2%) with no difference observed for iron supplementation during their last pregnancy (*p* = 0.328), lived in rural areas (64.7%) with a significantly higher percentage of women reported using iron supplementation in urban areas (45.8% vs. 35.3%; *p* < 0.001) compared to rural areas. The majority of women also reported not having smoked before becoming pregnant (98%), having fewer than four pregnancies (51.4%), no pregnancy loss (64.0%), fewer than four children (63.6%), with none of the children died (80%) with all the factors significantly associated with iron supplementation among women during pregnancy (*p* < 0.001). Further, women who had talked with a LHW regarding antenatal care made up 59.7% of the sample, with a higher percentage of women who had talked with an LHW (*p* < 0.001), got advice from LHW for referral of antenatal care (*p* < 0.005), and received antenatal care (*p* < 0.001) reported to take iron supplementation during pregnancy, which highlights the importance of uptake of antenatal healthcare services (Table 4).

### Predictors of iron supplementation during pregnancy among females of reproductive age

The results of regression indicate that women aged 36 and above are 0.68 times less likely to take iron supplementation during pregnancy compared to those under 25 years (OR = 0.68; 95% CI: 0.59–0.78). In contrast, women with secondary (OR = 1.62; 95% CI: 1.04–2.53), higher secondary (OR = 1.62; 95% CI: 1.00–2.63), and higher education (OR = 2.42; 95% CI: 1.49–3.91) are more likely to take iron supplements compared to those with less than primary education. Similarly, women residing in KPK (OR = 1.39; 95% CI: 1.16–1.55), GB (OR = 1.23; 95% CI: 1.01–1.49) and AJK (OR = 1.79; 95% CI: 1.47–2.18) are more likely to take iron supplementation during pregnancy than those in Punjab. However, women living in Sindh (OR = 0.74; 95% CI: 0.65–0.86) and Baluchistan (OR = 0.45; 95% CI: 0.39–0.53) are less likely to take iron supplementation compared to those resided in Punjab.

Additionally, women in rural areas (OR = 0.65; 95% CI: 0.58–0.71), those with a history of smoking (OR = 0.49; 95% CI: 0.36–0.69), those with four or more pregnancies (OR = 0.63; 95% CI: 0.57–0.69), those who have experienced pregnancy loss (OR = 0.68; 95% CI: 0.61–0.75), those with four or more children (OR = 0.63; 95% CI: 0.57–0.69), and those who have had a child die (OR = 0.73; 95% CI: 0.64–0.82) are also less likely to uptake iron supplementation during pregnancy.

Regarding institutional factors, consultation with LHW about antenatal care (OR = 1.58; 95% CI: 1.27–1.97), referral from LHW for antenatal care (OR = 1.46; 95% CI: 1.14–1.86) and receiving antenatal care (OR = 30.07; 95% CI: 23.55–38.40) significantly influence the likelihood of taking iron supplementation during pregnancy among Pakistani women of reproductive age (Table 4). The large CI of receiving the antenatal care highlights the large effect size and the importance of utilization of antenatal care and healthcare services.

When all the individual-level personal variables (age, education and smoking history) were collectively added, the effects of age and smoking history became insignificant while the education remained a significant predictor. Women with secondary (AOR = 1.62; 95% CI: 1.03–2.51); higher secondary (AOR = 1.87; 95% CI: 1.16–3.01) and higher education (AOR = 2.92; 95% CI: 1.82–4.69) were more likely to uptake iron supplementation during pregnancy. These findings signify that education is a stronger predictor of iron supplementation among women during pregnancy compared to age and smoking history.

In model 2, when both individual-level personal (age, education and smoking history) and obstetrics variables (total number of pregnancies, pregnancies lost, total number of children ever born and children ever died) were included, age and smoking history remained insignificant. However, higher secondary (AOR = 1.69; 95% CI: 1.04–2.75) and higher education (AOR = 2.55; 95% CI: 1.56–4.15) remained significant, while secondary education became insignificant. Among obstetrics variables, only pregnancy loss remained a significant predictor, with women who had experienced pregnancy loss being less likely to take iron supplementation (AOR = 0.48; 95% CI: 0.40–0.57) while total pregnancies, total children ever born, and child mortality became insignificant. In model 3, when individual-level personal, individual-level obstetrics and community level variables (type of residence and region of residence) were included, the effect of higher secondary education became insignificant. However, higher education (AOR = 2.16; 95% CI = 1.31–3.55) and pregnancy loss (AOR = 0.48; 95% CI: 0.40–0.57) remained significant. Women residing in KPK (AOR = 1.64; 95% CI: 1.27–2.12), GB (AOR = 1.54; 95% CI: 1.15–2.08), and AJK (AOR = 1.57; 95% CI: 1.24–2.00) were significantly more likely to take iron supplementation, while those in Baluchistan (AOR = 0.49; 95% CI: 0.37–0.66) were significantly less likely. Age, smoking history, total number of pregnancies, total number of children ever born and child mortality remained insignificant. Lastly, in model 4, when all the variables, including institutional level factors (LHW talked about antenatal care, LHW provided advise: referral for antenatal care, and received antenatal care for pregnancy), were included, only residing in KP (AOR = 2.21; 95% CI: 1.30–3.77), GB (AOR = 3.23; 95% CI: 1.58–6.60) and AJK (AOR = 1.68; 95% CI: 1.02–2.78), pregnancy loss (AOR = 0.45; 95% CI: 0.31–0.65) and receiving antenatal care (AOR = 31.29; 95% CI: 14.37–68.11) remained significant predictors of iron supplementation during pregnancy. All other variables became insignificant. Notably, women who receive antenatal care are 31.29 times more likely to uptake iron supplementation during pregnancy (Table 5). These findings highlight the large effect size of receiving antenatal care on the uptake of iron supplementation.

**Table 5:** Hierarchical regression model for predictors of iron supplementation during pregnancy.

Variables	Model 0	Model 1	Model 2	Model 3	Model 4
	OR (95% CI)	AOR (95% CI)	AOR (95% CI)	AOR (95% CI)	AOR (95% CI)
**Ag**e					
Under 25 years	1	1	1	1	1
26–35 years	0.96 (0.86–1.08)	0.93 (0.79–1.10)	1.01 (0.84–1.22)	1.01 (0.84–1.22)	1.21 (0.81–1.81)
36 years and above	0.68 (0.59–0.78)*	0.87 (0.68–1.12)	1.03 (0.77–1.37)	0.97 (0.72–1.29)	1.51 (0.80–2.85)
**Education**					
Less than primary education	1	1	1	1	1
Primary education	0.95 (0.61–1.47)	0.94 (0.61–1.46)	0.87 (0.56–1.36)	0.82 (0.53–1.29)	0.74 (0.28–1.96)
Middle education	1.39 (0.89–2.19)	1.38 (0.88–2.17)	1.25 (0.79–1.98)	1.11 (0.69–1.77)	0.80 (0.30–2.19)
Secondary education	1.62 (1.04–2.53)*	1.61 (1.03–2.51)*	1.46 (0.93–2.31)	1.30 (0.82–2.07)	1.25 (0.46–3.40)
Higher secondary	1.88 (1.17–3.02)*	1.87 (1.16–3.01)*	1.69 (1.04–2.75)*	1.51 (0.92–2.48)	1.10 (0.38–3.16)
Higher education	2.90 (1.81–4.64)*	2.92 (1.82–4.69)*	2.55 (1.56–4.15)*	2.16 (1.31–3.55)*	1.97 (0.66–5.88)
**Region of residence**					
Punjab	1			1	1
Sindh	0.74 (0.65–0.86)*			0.86 (0.68–1.08)	1.15 (0.67–1.97)
KPK	1.34 (1.16–1.55)*			1.64 (1.27–2.12)*	2.21 (1.30–3.77)*
Balochistan	0.45 (0.39–0.53)*			0.49 (0.37–0.66)*	1.05 (0.48–2.32)
GB	1.23 (1.01–1.49)*			1.54 (1.15–2.08)*	3.23 (1.58–6.60)*
AJK	1.79 (1.47–2.18)*			1.57 (1.24–2.00)*	1.68 (1.02–2.78)*
**Type of place of residence**					
Urban	1			1	1
Rural	0.65 (0.58–0.71)			0.81 (0.69–0.96)*	0.98 (0.70–1.38)
**Smoking before pregnancy**					
No	1	1	1	1	1
Yes	0.49 (0.36–0.69)*	0.83 (0.40–1.72)	0.84 (0.40–1.75)	1.43 (0.68–3.03)	1.17 (0.22–6.37)
**Total number of pregnancies**					
less than 4	1		1	1	1
4 and above	0.63 (0.57–0.69)*		1.04 (0.79–1.37)	1.06 (0.80–1.40)	0.92 (0.51–1.65)
**Pregnancy lost**					
No	1		1	1	1
Yes	0.68 (0.61–0.75)*		0.48 (0.40–0.57)*	0.48 (0.40–0.57)*	0.45 (0.31–0.65)*
**Total number of children ever born**					
Less than 4	1		1	1	1
4 and above	0.63 (0.57–0.69)*		0.94 (0.71–1.24)	0.96 (0.72–1.26)	0.80 (0.45–1.40)
**Children ever died**					
No	1		1	1	1
Yes	0.73 (0.64–0.82)*		1.12 (0.89–1.41)	1.14 (0.90–1.44)	0.89 (0.57–1.39)
**LHW talked about antenatal care**					
No	1				1
Yes	1.58 (1.27–1.97)*				1.18 (0.81–1.73)
**LHW provided advice: Referral for antenatal care**					
No	1				1
Yes	1.46 (1.14–1.86)*				1.07 (0.71–1.63)
**Received antenatal care for pregnancy**					
No	1				1
Yes	30.07 (23.55–38.40)*				31.29 (14.37–68.11)*

## Discussion

The present study found an increasing trend of iron supplementation during pregnancy among women of reproductive age in the country with a 44.1% increase nationwide, 61.7% increase in rural areas, and 19.9% increase in urban areas since 2006–07. The study further found that women of rural areas are 0.65 times less likely to uptake iron supplementation during pregnancy when compared to those living in urban areas. The present study found that despite of the increasing trend over the years, the rate of iron supplementation during pregnancy among women in the country stands at 65.4% which is lower than other South Asian countries including Bangladesh (81.0%), India (78.1%), and Nepal (77.0%)^36–38^ as well as other LMICs including Tanzania (81.6%) and Sub-Sahara Africa (79.7%)^39,40^. However, the rates of the country are relatively higher than that of Ethiopia (60.0%)^41^. Generally, it can be observed here that the rate of iron supplementation during pregnancy in Pakistan is lower as compared to other countries, however, the unswerving comparison of the findings of the present study with those conducted in other countries may not be possible because of the varying population dynamics, socio-cultural factors, and living conditions and variations in healthcare systems^42^. Differences in national policies, healthcare infrastructure, and maternal health awareness contribute to cross-country discrepancies in supplementation rates.

Further, the present study found a heterogeneity and disparity among the rates and trends of iron supplementation during pregnancy across different regions of the country, with the highest percentage increase observed in the region of GB (92.9%), followed by Punjab (59.6%) while the lowest increasing trend have been observed in Sindh with overall 59.5% prevalence rate of iron supplementation during pregnancy among women of reproductive age for the region for 2019 that stands at the second lowest after Baluchistan (47.3%). Further, the study documents 0.74 and 0.45 times less likelihood of dwellers of Sindh and Baluchistan and 1.23, 1.34, and 1.79 times more likelihood among women of GB, KP, and AJK to uptake iron supplementation during pregnancy as compared to those residing in Punjab. The disparities across different Pakistani regions, with particularly low rates in Sindh and Baluchistan compared to more substantial increases in Gilgit Baltistan, Punjab, and Khyber Pakhtunkhwa, point to the uneven impact of health interventions and the influence of socio-economic factors on health outcomes. These results should be interpreted in the light of region-specific population dynamics which show that around 46.3% of the population of Sindh is rural with 24.6% poverty rate in the region and that for Baluchistan is 69.0% and 40.7%, respectively, while the poverty rate of Punjab stands at 16.3%^43,44^. Further, women in Baluchistan face multiple barriers to accessing healthcare, including geographic remoteness, inadequate healthcare facilities, socio-cultural restrictions, and lower levels of maternal education^34^. Limited healthcare accessibility, particularly the shortage of LHWs and antenatal care services in rural Baluchistan, further exacerbates the issue. Additionally, socio-economic constraints and lower awareness levels contribute to poor adherence to supplementation recommendations. Addressing these disparities requires region-specific interventions, such as expanding healthcare access, increasing the number of trained healthcare workers, and enhancing culturally sensitive health education campaigns.

In addition to this, the present study found various underlying factors predicting iron supplementation during pregnancy among women in the country including age, education, level type of place of residence, smoking history, total number of pregnancies, pregnancy loss, total number of children ever born, children ever died, LHW advice and referral for antenatal care and attainment of antenatal care during pregnancy. Younger women’s higher likelihood of supplementation suggests a generational shift in health awareness and access to information, likely facilitated by digital media^33,45,46^.

Further, the present study found secondary, higher secondary, and higher education as the significant predictor for uptaking iron supplementation during pregnancy among women in Pakistan which resonates with the findings of many other similar studies conducted in Pakistan, Bangladesh, Malawi, Nigeria, Tanzania, Ethiopia, and many other sub-Saharan countries that report women having higher level of education to be more likely to consume iron supplementation during pregnancy^33,36,39,45,47–49^. It conceivably reflects that women who have a higher level of education are more knowledgeable, have higher access to information, and consequently can make more informed decisions regarding their health. Further, evidence also shows that educated mothers are more conscious about their own and upcoming child’s health and hold more healthcare decision-making autonomy as compared to non-educated mothers^42,50,51^. Women with higher education levels are more likely to adhere to supplementation recommendations, highlighting the need for initiatives that promote female education as a long-term strategy for improving maternal and child health outcomes.

The present study further found a negative relation between smoking history, total number of pregnancies, pregnancies loss, total number of children, and children ever died with iron supplementation during pregnancy among women of reproductive age. The relation between smoking history and iron supplementation could be alluded to the personal health-seeking attitudes while the negative association of different obstetric characteristics perhaps shows that a woman has experienced multiple uneventful pregnancies leading to lower to non-reliance on iron supplementation during pregnancy.

Lastly, with regard to institutional level factors, a positive relation has been found of LHW talked regarding antenatal care and LHW provided advice regarding antenatal care with attainment of antenatal care as the strongest predictor of iron supplementation during pregnancy increasing the likelihood of supplementation by 30.07 folds in unadjusted model and 31.29 folds in adjusted model. These findings resonate with the previous literature that found antenatal care and antenatal visits as the strongest predictor of iron supplementation during pregnancy and its compliance in different parts of the world^33,39,41,49^. Further, this association underscores the critical role of antenatal care in promoting maternal health behaviors. Antenatal visits serve as a platform for educating women on the benefits of iron supplementation, ensuring access to supplements, and reinforcing adherence through health worker counseling^34^. Policymakers should prioritize scaling up antenatal care services, particularly in underserved regions, to further enhance supplementation uptake and reduce maternal anemia.

Based on the highlighted factors and the overall findings of the study, there is a need to take necessary measures for improving the antenatal care coverage in the country. This is because in the primary healthcare system of Pakistan, antenatal care plays a major role for expanding the coverage of iron supplementation during pregnancy as it is responsible for providing free of cost iron supplementation to women during pregnancy, hence, revealed as the strongest predictor. The findings call for targeted strategies to address regional disparities, enhance education and awareness regarding maternal nutrition, and improve access to antenatal care. By focusing on these areas, policymakers and healthcare providers can make significant strides in increasing iron supplementation rates, contributing to healthier pregnancies and better neonatal outcomes across Pakistan.

### Strengths and limitations

The present study is one of its kinds that evaluates the predictors of iron supplementation during pregnancy among women of reproductive age as well as documents the trend of iron supplementation during pregnancy over the course of time using four rounds of PDHS data. The study summarizes the data of country-wide survey and generates national findings on it that could be used to provide pathway to policymakers and healthcare professionals for achieving optimal levels perinatal iron supplementation in the country. However, there are some limitations of the present study. Firstly, the present study utilizes secondary data which limits control over variable selection, potentially excluding relevant predictors. For instance, the findings of the predictors of iron supplementation are analyzed using PDHS 2019, which is a mini PDHS with unavailability of data on many variables including respondent’s occupation, husband’s education, husband’s occupation, age at marriage, age at first pregnancy, and questions pertaining to female autonomy which prevented to capture the effect of these predictors. The generalizability of the results may be influenced by unmeasured confounders or selection bias. Secondly, as PDHS data is collected through self-reporting from respondents, the responses may be subjected to recall and self-reporting bias. Lastly, the present study considers iron supplementation during pregnancy irrespective of the days, the supplementation had been used.

## Conclusions

Conclusively, the present study found a lower national as well as regional coverage of iron supplementation during pregnancy among women of reproductive age in comparison to other countries, despite of the increasing trend since 2006–07. The finding that access to antenatal care significantly influences supplementation uptake is particularly illuminating, pointing to the central role of comprehensive maternal healthcare services in improving iron supplementation rates. Significant regional disparities suggest the need for targeted efforts to increase supplementation rates and improve maternal health outcomes, such as increasing healthcare access in underperforming regions, expanding educational campaigns, strengthening community-based programs and enhancing the LHWs capacity to improve supplementation adherence.

Efforts to enhance iron supplementation should not only focus on expanding access to antenatal care but also on educating communities about the importance of maternal nutrition and the potential implications of iron deficiency for both mother and child. This dual approach requires collaboration across sectors, involving healthcare providers, policymakers, community leaders, and educational institutions to ensure that pregnant women receive the support they need to access and adhere to iron supplementation regimens. There is a need to develop a holistic understanding of all the underlying factors, with attainment of antenatal care as the strongest predictor, to develop more effective strategies and to design concerted efforts for achieving higher coverage of iron supplementation across the country by reducing the regional disparities.

## Data Availability

All PDHS data is publicly available: https://dhsprogram.com/data/available-datasets.cfm.

## Abbreviations

AJK: Azaad Jammu Kashmir
AOR: Adjusted odds ratio
DALY: Disability-adjusted life year
CI: Confidence Interval
GB: Gilgit Baltistan
ICT: Islamabad Capital Territory
KPK: Khyber Pakhtunkhwa
LMIC: Low- and-middle-income country
LHW: Lady health worker
OR: Odds ratio
PDHS: Pakistan Demographic and Health Survey
WHO: World Health Organization
